# Schistosomiasis (*Schistosoma mansoni*)—a rare cause of complex liver cysts

**DOI:** 10.1093/jscr/rjad452

**Published:** 2023-08-08

**Authors:** Jan Chmielewski, Pascal Probst, Markus K Muller, Pia Antony, Dragoljub Kovacevic

**Affiliations:** Department of Surgery, Cantonal Hospital Thurgau, 8501 Frauenfeld, Switzerland; Department of Surgery, Cantonal Hospital Thurgau, 8501 Frauenfeld, Switzerland; Department of Surgery, Cantonal Hospital Thurgau, 8501 Frauenfeld, Switzerland; Department of Surgery, Cantonal Hospital Thurgau, 8501 Frauenfeld, Switzerland; Department of Surgery, Cantonal Hospital Thurgau, 8501 Frauenfeld, Switzerland

## Abstract

In this paper, we describe the case of a 40-year-old patient with an expanding and symptomatic complex liver cyst. Despite comprehensive diagnostics, including labs, imaging and biopsy, a clear etiology could not be determined. As a result, a partial liver resection was performed. The histopathological examination revealed evidence of schistosomas. We postulate that the displacement of the portal fields created a pseudocyst and that the resultant ischemia was the root cause of the patient’s discomfort. Postoperatively, the patient received an antihelmintic therapy with praziquantel with which she was able to fully recover.

## INTRODUCTION

Schistosomiasis is one of the most prevalent parasitoses, with an overall prevalence of ~240 million patients worldwide. Though preventative measures in the last 20 years have been able to significantly reduce the incidence by almost 60% (especially in children), ~700 million people reside in the endemic regions of sub-Saharan Africa, South America and parts of Asia [1].

Due to increasing globalization, however, cases in western Europe have also become more common [2]. In fact, current S1-guidelines recommend screening in asymptomatic travelers returning from the above mentioned tropical climates [3].

Parasitosis is caused by infection from blood flukes. As a miracidium, they multiply and mature in fresh water snails to cercariae, who, in turn, swim to penetrate their human host. Afterwards, they enter the blood stream and complete maturation within the portal vasculature. Finally, paired adult schistosomas travel to target organs, the mesenteric venules of the bowel or the venous plexus of the bladder where they then lay their eggs [4].

Clinical presentation is varied and depends on the type and localization of the schistosoma. With hepatic manifestation, patients present with hepatomegaly and periportal fibrosis that may lead, depending on the degree of fibrosis, to portal hypertension. Ultrasound studies show a broadening of the periportal border zones. An increase in serum transaminases or other liver function parameters is not generally observed [5].

## CASE REPORT

**Figure 1:**
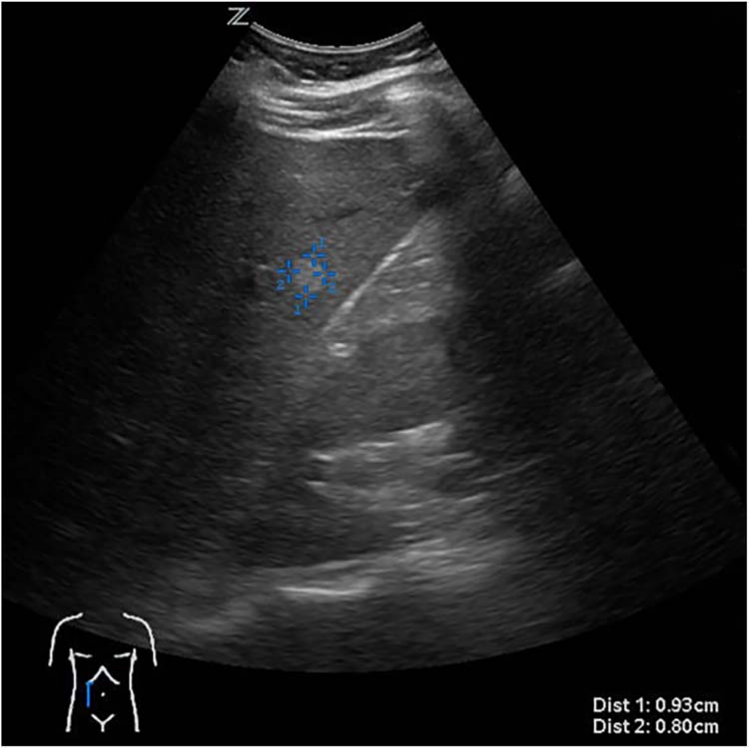
First documentation of liver cyst of the patient in ultrasound.

**Figure 2:**
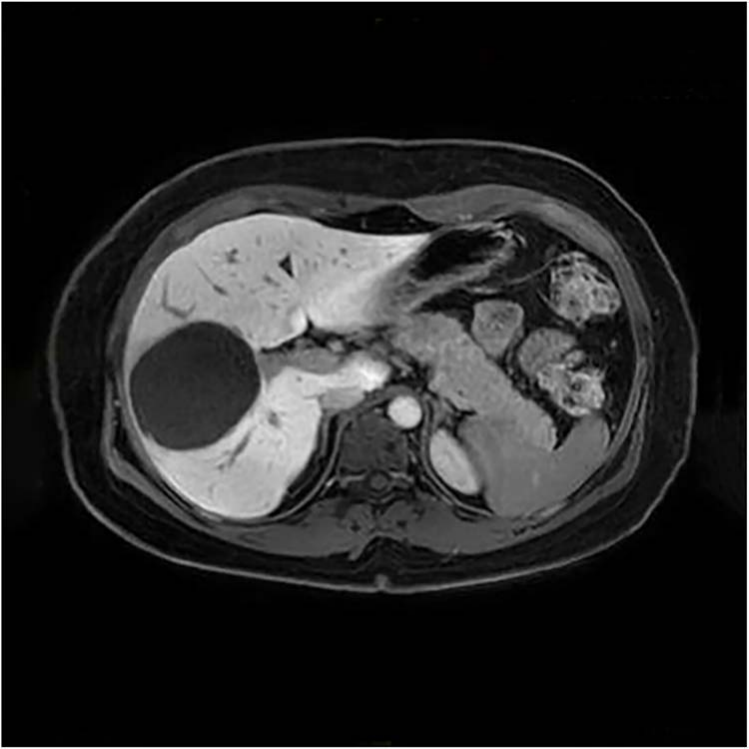
Evidence of a size-progressive septated liver cyst in the MRI.

The presenting patient was a 40-year-old Brazilian native who had lived in Europe for **~**26 years. Initially, the patient presented to her general practitioner with upper right quadrant pain. An ultrasound study (Fig. 1) showed a small hyperechoic lesion in segment VII measuring **~**0.8 × 0.9 cm. Follow-up studies showed an expanding lesion with newer hyperechoic portions. In 2020 a liver specific magnetic resonance imaging (MRI) (Fig. 2) was performed. Here, again, the cyst was expanding, septated and had reached a size of 7.3 × 6 × 6.5 cm. A Echinococcus infection was considered, however, repetitive EIisa for Echinococcus sp./IgG was negative. The alpha protein was 2 ug/L, and well within normal limits. Due to increasing discomfort, an upper gastrointestinal tract endoscopy was performed. Here, gastritis and gastro-intestinal neoplasia could be excluded.

**Figure 3:**
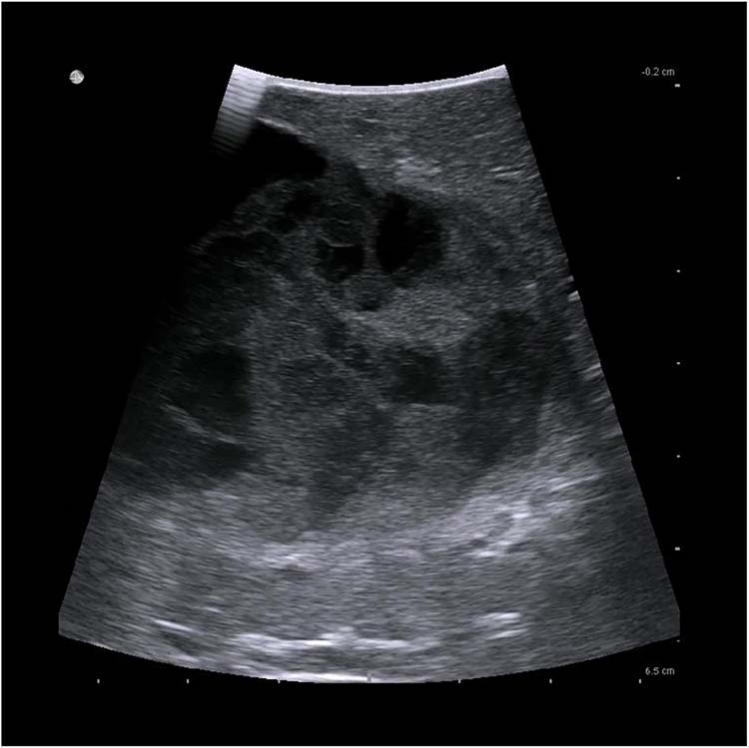
Intraoperative ultrasonographic localization of the liver cyst.

Due to persistent pain, the decision was made to perform a laparoscopic cyst deroofing. In an intraoperatively performed ultra sound (Fig. 3), however, a complex septation was seen so that the decision was made to perform a tru-cut biopsy in order to definitively rule out malignancy.

Unfortunately, histopathological examination showed only chronic inflammation with fibrosis. No evidence of malignant transformation was noted. Even in the postoperatively performed follow-up MRI, no malignant criteria could be identified. However, as a result of the extreme discomfort felt by the patient as well as the still unclear nature of the lesion, the decision was made to perform a partial en bloc liver resection of segments V, VI and VII.

**Figure 4:**
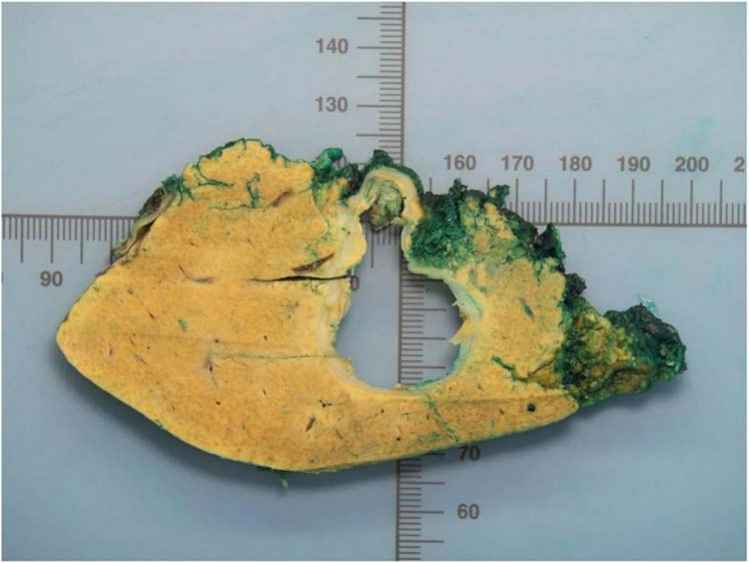
Macroskopic findings of the liver resection.

**Figure 5:**
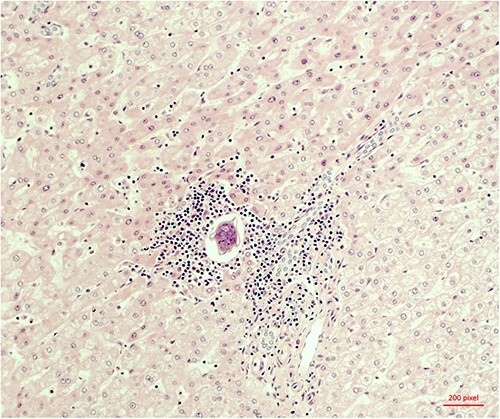
Microscopic evidence of *Schistosoma mansoni.*

The histopathological work-up revealed the already described macroscopic cystic lesion (Fig. 4). More precisely, a pseudocyst, which differs from a cyst in that it does not possess an epithelial lining. Within the pseudocyst, necrotic liver parenchyma was found. In addition, and more surprisingly, parasites (Fig. 5) were found within the portal fields, partially within the blood vessels. These appeared to be schistosomas. It appeared that the necrosis within the pseudocyst was formed pursuant to ischemia. Finally, proof of schistosomiasis mansoni eggs in stool cultures was able to definitively render the diagnosis. An antihelmintic therapy with praziquantel was begun, under which the patient was able to completely recover.

## DISCUSSION

Due to the increasing frequency of imaging diagnostics, the number of incidentally diagnosed liver cysts has increased greatly [6]. Studies show that the prevalence of liver cysts in computed tomography scans is **~**18% [7]. Affected patients are often disquieted and request further diagnostics.

In addition to simple cysts, further cystic lesions include biliary cystadenomas/adenocarcinomas, polycystic liver diseases, caroli syndrome or echincoccus can be identified by radiologic imaging.

From a therapeutic stand point, simple asymptomatic cysts require no intervention. Symptomatic cysts, by contrast, can generally be adequately treated with laparoscopic deroofing, thereby minimizing the need for resection [8]. Indeed, among the more frequent indications for resection of liver cysts is uncertainty in the preoperative diagnostics [9].

The case we describe here presents yet another differential diagnosis. We would, however, like to point out, that during the course of our research no other cases of cystic liver lesions due to a Schistosoma’s infection were identified. The diagnosis of schistosomas is possible through a variety of means, so that surgical resection would not generally be necessary. For example, diagnosis can be secured through urine or stool samples, as well as point-of-care tests searching for circulating antibodies (POC-CCA/CCA) from urine or serum.

Schistosomiasis is usually treated with a short course of oral antihelmintic therapy, usually praziquantel for 1–3 days. Avital parasite eggs, however, will still be excreted over a period of several months. Therefore, follow-up tests after 6, 12 and 24 months are recommended [3]. Because only individual isolated cases have been reported in central Europe, mostly likely due to infections acquired abroad, no epidemiological data in Switzerland is available. Despite this, schistosomas has become increasingly relevant due to expanding endemic regions. Indeed, since 2014 the endemic region has expanded to include southern corsica; the first within Europe. In addition, increased travel to other endemic regions will undoubtedly further lead to an increase in cases in central Europe. This is especially important because a correlation between S. hämatobium and squamous cell carcinoma of the urogenital tract, as well as an indirect association between *Schistosoma mansoni* and hepatocellular cancer is probable [10].

## Data Availability

Authors confirm that any required links or identifiers are present in the manuscript.
